# Association of Myo-Inositol and Microlipodispersed Magnesium in Androgen-Dependent Dermatological Diseases: A Retrospective Study

**DOI:** 10.3390/ph18020251

**Published:** 2025-02-13

**Authors:** Michele Pezza, Valentina Carlomagno, Elena Sammarco, Antonino Trischitta, Carla Ceddia, Amalia Vitiello, Germano Baj, Valentina Citi, Alessandro Colletti

**Affiliations:** 1 Dermatological Surgery-Melanoma Villa Maria, 83036 Passo di Mirabella Eclano, Italy; 2 Ditar Dermatological Laser Therapy Center, 82100 Benevento, Italy; 3 Hospital Company of National Importance (AORN) Santobono-Pausilipon, 80123 Naples, Italy; 4 Outpatient Local Health Board (ASL) Napoli 2 Nord, 80027 Naples, Italy; 5 ADECA (Campania Dermatologists Association), 80124 Naples, Italy; 6 Applied Studies Center for Medicinal Herbs and Minor Fruits (CAOM), 28100 Novara, Italy; 7 Department of Pharmacy, University of Pisa, 56126 Pisa, Italy; 8 Department of Drug Science and Technology, University of Turin, 10125 Turin, Italy

**Keywords:** myo-inositol, acne, PCOS, magnesium, hirsutism, folic acid

## Abstract

**Background:** Acne is a pathology of the pilosebaceous unit. It is characterized by a highly complex etiopathology which includes inflammation, hyperkeratinization, increased sebum production, colonization of *Cutibacterium acne*, hyperandrogenemia, and hyperinsulinemia. This condition, together with hirsutism, androgenic alopecia, and acanthosis nigricans, are highly prevalent cutaneous manifestations of the polycystic ovary syndrome (PCOS). While conventional therapies represent effective treatment options, they are not free from side effects which may reduce compliance. In this context, considerable attention has been directed toward nutraceutical supplements, which include different molecules with great potential to reduce inflammation, hyperkeratinization, hyperseborrhea, and hyperinsulinemia. Myo-inositol has been shown to be effective in improving some of the signs and symptoms of patients with microcystic ovaries: reducing body mass index (BMI), testosterone free levels, dehydroepiandrosterone sulfate (DHEAS) levels, and improving ovarian function and insulin sensitivity. **Methods**: The authors conducted a retrospective study that included 200 patients suffering from PCOS. Over 6 months, they analyzed the effects of the supplementation of LEVIGON™ (Sanitpharma; Milan, Italy)—a specific nutraceutical formulation containing myo-inositol, microlipodispersed magnesium, and folic acid—on the clinical picture of acne and hirsutism. **Results**: The supplementation of LEVIGON™ showed a significant reduction of BMI, testosterone, testosterone free, and DHEAS levels, thus improving the clinical picture of acne and hirsutism. Moreover, the impact of acne on the quality of life, assessed using the Cardiff Acne Disability Index (CADI) and Dermatology Life Quality Index (DLQI) scale, improved significantly after 3 and 6 months. Women with hirsutism benefited also from a significant improvement of the Ferriman-Gallwey score after both 3 and 6 months (*p* < 0.0001; *p* < 0.0001 respectively compared to the baseline). **Conclusions**: Myo-inositol supplementation, associated with microlipodispersed magnesium in a bioaccessible form, proved to be extremely useful in reducing acne and hirsutism in patients suffering from microcystic ovaries. In addition, there were no side effects, thus confirming excellent compliance. Further long-term randomized clinical trials are needed to confirm this preliminary evidence.

## 1. Introduction

**Figure 1:**
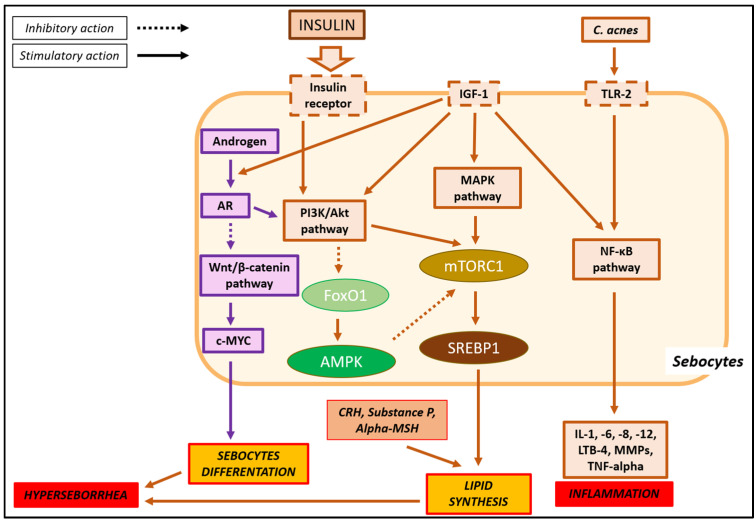
Etiopathological role of insulin resistance in acne.

Polycystic ovary syndrome (PCOS) is well recognized as a multifactorial genetically complex endocrine disorder, characterized by an increasing prevalence among women of all age groups [1]. PCOS affects 4–20% of women of reproductive age worldwide [2], and it includes a wide range of clinical implications, such as reproductive dysfunctions (pregnancy complications, infertility, menstrual irregularities, and hirsutism), cardiovascular risk factors (e.g., dyslipidemia), metabolic dysregulations (impaired glucose tolerance, insulin resistance, type 2 diabetes, and metabolic syndrome), oncological complications (endometrial, ovarian and breast cancers), and psychological implications (negative body image, depression, anxiety, and worsened quality of life) [3]. A metanalysis, which included data from 60 studies (240,213 women with PCOS and 1,902,022 healthy controls), showed an overall pooled prevalence of acne among women with and without PCOS of approximately 43% and 21%, respectively, confirming that acne is one of the common manifestations of the PCOS [4]. In this context, insulin resistance (IR) plays a fundamental role in the development of both PCOS and acne (Figure 1), with a prevalence between 35% and 80% in PCOS women [5]. PCOS women with IR may present with impaired fasting plasma glucose levels, reduced responsiveness or sensitivity to the metabolic actions of insulin, and chronic subclinical inflammation. These are known to be closely associated with a range of disorders, such as cardiovascular and skin diseases [6]. Given the high prevalence of IR in women with PCOS, early recognition, management, and accurate screening are necessary to offer important preventive measures [6].

Lifestyle changes represent the first-line intervention measures in the management of PCOS. These actions enable improved insulin sensitivity, and include an increase in physical activity (specifying the number of times per week, type of activity, and duration), a caloric intake proportional to the individual's energy needs, the correct balance of macro and micronutrients (reducing the content of saturated and trans esterified fats, and sugars), and weight reduction in overweight or obese subjects [7]. In recent years, different nutraceutical compounds have demonstrated an improvement in the clinical signs and symptoms of PCOS, and the associated *Acne vulgaris* [8]. Particularly promising are the randomized clinical trials (RCTs) that demonstrate the usefulness of myo-inositol in the treatment of PCOS [3]. The meta-analysis by Greff et al. (26 RCTs and 1691 patients) showed that, in subjects treated with inositols, the chance of having a regular menstrual cycle was found to be 1.79 higher than for subjects treated with a placebo, thus improving the clinical manifestations of PCOS, such as IR, hyperandrogenism, fertility, and oligoamenorrhea [9].

Myo-inositol (C_6_H_12_O_6_, hexahydroxy cyclohexane) is a sugar alcohol or polyol that is naturally present in a variety of foods, such as beans, fruits, vegetables, almonds, and walnuts [10]. Myo-inositol forms an important component of membrane lipids. It mediates the signal transduction in response to different hormonal stimuli, growth factors, and neurotransmitters, thus playing a fundamental role in the efficiency of several cellular functions (e.g., regeneration and conduction of peripheral nerves, smooth muscles, osteogenesis, and reproduction) [11]. In this regard, biological molecules, such as inositol phosphates (IP3), phosphatidylinositol phosphate lipids (PIP2/PIP3), and inositol glycans (IPGs), contain myo-inositol as a component, and they act as second messengers for numerous cellular activities [12]. 

Myo-inositol participates in both insulin signaling and glucose metabolism by influencing distinct pathways and decreasing IR that represents the cornerstone in the development of various metabolic dysfunctions in PCOS [13]. This molecule induces glucose transporters (GLUT) translocation to the cell membrane, enhancing the cellular uptake of glucose. It also stimulates the pyruvate dehydrogenase complex (PDH) (supporting ATP production through the Krebs cycle), and the glycogen synthase (GS) (supporting glucose conversion to glycogen stored inside cells), and its derivatives inhibit the adenylate cyclase (ADC) enzyme reducing free fatty acids (FFAs) released from the adipose tissue: beneficial because elevated concentrations of FFAs cause impairment of intracellular insulin signalling) [14]. In this regard, the deficiency of myo-inositol seems to play a key role in the development of IR in PCOS. It has been observed that, in patients with PCOS, there is an increased urinary excretion of inositols leading to its deficiency [3]. The possible mechanisms for elevated urinary excretion of inositols in people with IR include reduced a renal threshold for D-chiro-inositol, an abnormal tissue/cellular uptake of inositols, and/or abnormal intracellular processing of inositols (e.g., myo-inositol conversion to D-chiro-inositol) [15].

In this context, the present retrospective analysis aimed to determine the effect of a specific formulation containing myo-inositol, magnesium, and folic acid in the treatment of acne in women with PCOS. 

## 2. Results

Two hundred and fifty-seven patients were screened, of which two hundred completed the study. Fourteen patients were excluded due to concomitant pathologies (thyroid disease and/or diabetes type II) and seventeen patients were excluded due to the concomitant use of drugs. Twenty-six patients were lost in the follow up. One hundred and forty patients had acne and sixty presented with acne and hirsutism. The average age of the patients enrolled was 25.4 years. Patients were evaluated at time zero (T0), and after 3 (T1) and 6 months (T2) of supplementation. All patients took two grams of myo-inositol in addition to folic acid (200 mcg/day) and microlipodispersed magnesium (56.25 mg/day), two times a day for six months.

Already at 3 months (T1) the intake of LEVIGON^™^ had significantly reduced DHEAS (251 ± 39 ng/dL; 95.00% CI of diff. [123.4–142.6]; *p* < 0.0001), testosterone (79 ± 29 ng/dL; 95.00% CI of diff. [17.4–30.6]; *p* < 0.0001), and free testosterone (0.8 ± 0.3 ng/dL; 95.00% CI of diff. [0.45–0.55]; *p* < 0.0001). In addition, a significant reduction of DHEAS (167 ± 31 ng/dL; 95.00% CI of diff. [207.4–226.6]; *p* < 0.0001), testosterone (59 ± 24 ng/dL; 95.00% CI of diff. [37.4–50.6]; *p* < 0.0001), and free testosterone (0.6 ± 0.2 ng/dL; 95.00% CI of diff. [0.64–0.75]; *p* < 0.0001) was observed after 6 months (T2) of treatment (Table 1). GEA showed an improvement of the clinical picture of acne after 3 months of treatment (2.1 ± 0.9; 95.00% CI of diff. [1.1–1.5]; *p* < 0.0001), with a further decrease in the severity of acne signs at 6 months (0.7 ± 0.6; 95.00% CI of diff. [2.4–2.9]; *p* < 0.0001) (Table 1). 

Basal insulin and Homeostasis Model Assessment (HOMA) index were improved significantly after 3 months of treatment (respectively, 22 ± 3.9 mcIU/mL; 95.00% CI of diff. [2.2–3.8], *p* < 0.0001; 1.8 ± 0.6; 95.00% CI of diff. [0.9–1.2], *p* < 0.0001). The improvement was maintained also in T2, where Basal insulin and HOMA index were respectively 17 ± 2.8 mcIU/mL; 95.00% CI of diff. [7.2–8.8] *p* < 0.0001 and 1.4 ± 0.4; 95.00% CI of diff. [1.4–1.6] *p* < 0.0001 (Table 1).

The impact of acne on quality of life, assessed using the CADI scale, reduced from an initial average score of 9.2 ± 1.7 (T0) to a final average score of 2.9 ± 0.9 (T2); 95.00% CI of diff. [6.1–6.6] (*p* < 0.0001). Even the DLQI improved from 9.8 ± 1.7 (average baseline values) to 2.0 ± 0.8; 95.00% CI of diff. [7.5–8.1] (*p* < 0.0001). Patients with hirsutism benefited from a significant improvement of Ferriman-Gallwey score after both 3- and 6-months 12.1 ± 2.1; 95.00% CI of diff. [1.2–3.2] *p* < 0.0001; 8.4 ± 1.2; 95.00% CI of diff. [4.8–6.9] *p* < 0.0001, respectively compared to baseline) (Table 2).

BMI was significantly reduced after 3 months of treatment compared with the BMI evaluated before starting the treatment (25.7 ± 2.9; 95.00% CI of diff. [0.14–1.4]). The reduction was even more marked after 6 months of treatment (T2) reaching 23.9 ± 2.8; 95.00% CI of diff. [1.9–3.3] as reported in Table 3.

LH and FSH levels were significantly reduced after 3 months of treatment (T1) compared to baseline, respectively 10.5 ± 3.6 mIU/mL; 95.00% CI of diff. [3.7–5.4] *p* < 0.0001 and 5.5 ± 1.5 mIU/mL; 95.00% CI of diff. [0.26–0.93] *p* < 0.0001). This effect was more significant after 6 months (T2) where LH and FSH reached respectively 8.3 ± 2.2 mIU/mL; 95.00% CI of diff. [5.9–7.6] *p* < 0.0001 and 4.4 ± 1.3 mIU/mL; 95.00% CI of diff. [1.3–2.04] *p* < 0.0001 (Table 1).

No side effects have been reported through the diary sheet of patients and compliance was excellent.

## 3. Discussion

PCOS is a condition characterized by chronic anovulation, an excess of androgen production, and it is frequently associated with different hormonal diseases which can cause acne, hirsutism, seborrhea, and pattern alopecia [16]. Given the important role of IR in the pathophysiology of both acne and PCOS, there is the plausibility of the rationale for IR treatment in women with PCOS with the goal of reducing the metabolic disorders and promoting the normalization of ovulatory function [17]. The American Association of Clinical Endocrinologists recommends metformin as the initial step in most women with the PCOS, especially those who are overweight or obese [18]. The meta-analysis by Yen et al., which included 51 studies and 2405 PCOS women, highlighted that metformin, used in addition to conventional therapies, led to greater improvement of acne scores if compared with the same therapies without metformin, and the presence of acne decreased significantly after metformin treatment [19]. However, a meta-analysis of 71 RCTs and 55,042 patients showed that metformin use was associated with a high probability of an occurrence of diarrhea, abdominal pain, and nausea compared with the use of a placebo [20]. The risk of gastrointestinal side effects may be associated with a reduction of the compliance and an increased risk of treatment failures. Metformin could be associated also with the reduction of serum levels of vitamin B12, increasing the risk of hyperhomocysteinemia which is highly prevalent in the female population [21]. It seems that metformin may give a positive charge to the membrane’s surface of the cubilin receptor, causing a possible vitamin B12 malabsorption because the calcium-dependent binding of the intrinsic factor-vitamin B12 complex to the ileal cubilin receptor is compromised [22]. Finally, the safety of metformin in pregnancy remains unclear and should also be investigated to examine the mechanisms linking metformin to obesity during childhood, reported in some follow-ups [23,24]. In this context, myo-inositol has been suggested to improve IR in women with different dermatological [25,26], urological [27], and gynaecological [28] conditions, maintaining an excellent safety profile. 

**Figure 2:**
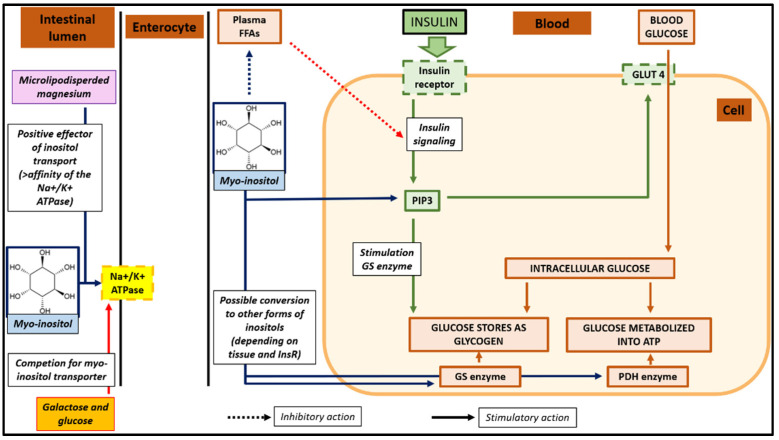
Intestinal absorption of myo-inositol, role of magnesium as positive effector of inositol transport, and myo-inositol effects on insulin resistance.

Myo-inositol is transported and absorbed into intestinal cells through the active transport system involving the Na^+^/K^+^-ATPase. In this context, magnesium is a positive effector of myo-inositol transport, promoting a 2.5-fold increase in the affinity of the Na^+^/K^+^-ATPase transporter for myo-inositol (Figure 2) [29]. However, while inorganic salts such as magnesium oxide provide a high loading of elemental magnesium, they exhibit a very limited bioaccesibility due to their poor solubility, potentially compromising their action as modulators of the Na^+^/K^+^-ATPase transporter [30]. In this regard, the use of a microlipodispersed system, characterized by a specific blend of the mono- and diglycerides of fatty acids, has been shown to improve the enteric bioaccessibility of inorganic forms of magnesium [31].

Galactose, glucose, and other sugars reduce the intestinal absorption and kidney reabsorption of myo-inositol. Elevated blood glucose and insulin resistance depletes myo-inositol levels in tissues due to a competitive inhibition of the Na^+^/K^+^-ATPase transporter [32]. For this reason, the supplementation of myo-inositol was recommended in a fasted state.

In recent years, the combination of myo-inositol with D-chiro-inositol in different ratios (40:1, 80:1) has been proposed for the co-management of PCOS [33]. However, the rationale for this combination remains unclear and needs further investigation, since D-chiro-inositol seems to inhibit myo-inositol absorption, decreasing its plasma concentration as compared to myo-inositol alone [34]. In addition, D-chiro-inositol acts as an aromatase down-modulator, with a still unknown mechanism of action, causing a possible increase in androgens and a decrease in estrogens [35]. Moreover, the observed pictures of toxicity for ovarian histology and function recommend caution when D-chiro-inositol is supplemented at high doses (>2 g/day) to PCOS patients [36].

Different formulations available on the market exploit the combination with other molecules that promote myo-inositol absorption, including the association with α-lactalbumin. This is a protein, naturally found in mammalian milk, that has a role in improving myo-inositol bioavailability, probably due to changes in tight junction permeability [37]. However, the alterations of intestinal permeability may not guarantee a selective improvement in the absorption of myo-inositol, but also of other substances such as lipopolysaccharide (LPS) and microbiome-derived endotoxins, causing a chronic low-grade pro-inflammatory condition named metabolic endotoxaemia, that could potentially worsen the clinical picture of the acne patient [38]. For this reason, the real benefit of long-term supplementations of myo-inositol with permeability enhancers such as α-lactalbumin remains unclear, which is further complicated by the well-known potential allergenicity of this ingredient [39]. 

The present retrospective study showed that the supplementation for 6 months of a specific formulation based on myo-inositol, microlipodispersed magnesium, and folic acid improved the clinical picture of acne and hirsutism in women with PCOS. Moreover, myo-inositol treatment decreases circulating androgen levels and improves insulinemia and HOMA index. These effects are in agreement with the meta-analysis by Greff et al. (26 RCTs and 1691 patients) [18]. However, knowing the metabolic derangements associated with PCOS, it seems reasonable to recommend a long-period supplementation that addresses the new goals of improving IR and reducing the risks of diabetes type 2 and cardiometabolic disease, and not simply managing the consequences of androgen excess and anovulation. In this context, insulin-sensitizer drugs represent novel options for dermatological disorders in subjects with PCOS, who do not need or desire contraception. Myo-inositol, through the amelioration of the insulin signal and consequently the reduction of insulin levels, represents an effective and safe treatment.

The combination of myo-inositol with magnesium in the formulation produces an improvement in the affinity of the Na^+^/K^+^-ATPase transporter for myo-inositol, potentially increasing its effectiveness. Studies conducted to date showed that many women with PCOS also exhibit hyperhomocysteinemia [40]. In this regard, the supplementation of folic acid significantly decreased homocysteine levels [41] and has been proposed in association with myo-inositol.

Our study has some limitations. First, this is a retrospective study with a relatively small number of cases; further double blind, randomized, controlled clinical trials will confirm this preliminary evidence. Second, the limited hormonal and metabolic factors included in the analysis were from “clinical practice”. Furthermore, the period of treatment was relatively short; the observed effect should also be confirmed for long-term treatment periods. However, since the myo-inositol mechanisms of action are similar as those of metformin, a drug that has extensively proven to maintain its efficacy over decades, we could reasonably hypothesize that this evidence may be translated to the product tested. 

## 4. Materials and Methods

### 4.1. Study Design and Participants

**Figure 3:**
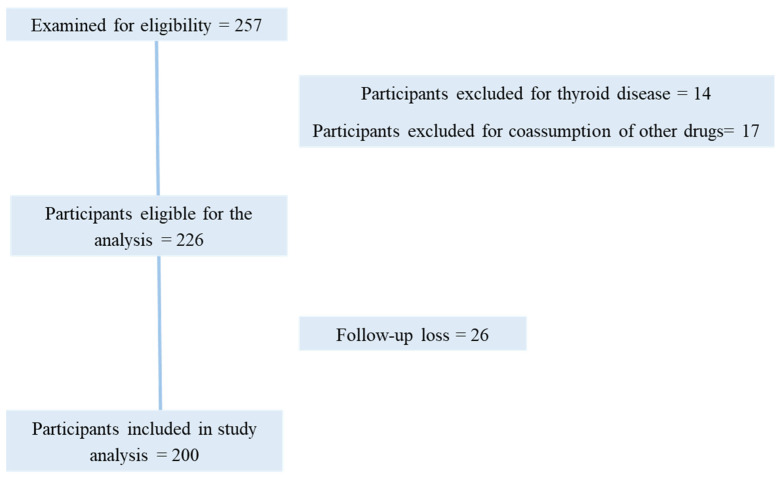
Flowchart of participants in the study.

This was a retrospective study which involved 200 female patients (Figure 3) aged between 20 and 35 years, suffering from acne, and diagnosed with microcystic ovaries. Females were treated with a specific formulation that included myo-inositol, magnesium, and folic acid, for six months and were retrospectively analyzed. 

The inclusion criteria of the retrospective analysis concerned the following:

(1) Female patients aged between 20 and 35 years.
(2) Diagnosis of ovarian polycystosis carried out with two of the following three criteria established by the Consensus of the European Society of Human Reproduction and Embryology [ESHRE]/American Society of Reproductive Medicine [ASRM]:
  - Oligoanovularity, with irregularities of the menstrual cycle.
  - Elevated circulating levels of androgens or clinical manifestations of hyperandrogenism.
  - Evidence of ovarian micropolycystosis on pelvic ultrasound.

Exclusion criteria encompassed the presence of other endocrinological diseases or any medical or surgical conditions that could influence the results. Additionally, exclusion criteria included concurrent use of other supplements. Patients were excluded from this analysis if they had incomplete data or missed scheduled visits, or if they reported the use of non-trial drugs during the treatment with nutraceutical.

Participants received the treatment for the first 3 months. Patients were evaluated at time zero (T0), after 3 months (T1), and after 6 months of supplementation (T2) for clinical status, in addition to being evaluated for compliance and the tolerability of the nutraceutical product. At each visit the body mass index (BMI), hormonal (LH, FSH, testosterone, free testosterone, DHEAS) and metabolic profile (basal insulin, HOMA index), Global Acne Severity (GEA) Scale, Dermatology Life Quality Index (DLQI), Cardiff Acne Disability Index (CADI), and hirsutism through the Ferriman and Gallwey scoring system were evaluated.

Informed written consent for the use of personal data for the present study was obtained from all the participants. 

**Figure 4:**
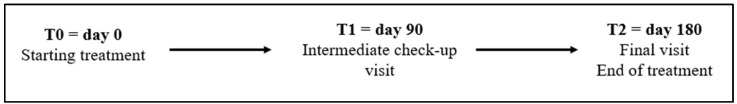
Study timeline.

The timeline of the study is described in detail in Figure 4.

### 4.2. Treatment

Each patient was treated with LEVIGON^™^ (Sanitpharma; Milan, Italy) at a dosage of 2 sticks twice a day for six months (T0–T2). LEVIGON^™^ is a patent formulation that contains 2 g of myo-inositol, 56.25 mg of microlipodispersed magnesium, and 200 mcg of folic acid. Throughout the entire period of treatment, patients were directed to take the designated supplement at approximately the same time each day, in fasted state. 

### 4.3. Efficacy Assessment

The primary outcome of this study was to analyze the use of LEVIGON™, at a dosage of 2 sticks twice a day for six months, in females suffering from acne, and diagnosed with microcystic ovaries (diagnosis made according to the criteria established by Consensus of the European Society of Human Reproduction and Embryology [ESHRE]/American Society of Reproductive Medicine [ASRM]) [42]. In this context, GEA scale (Table 4) is a validated global tool that can be used by clinical researchers to evaluate the severity of acne [43]. In addition, Dermatology Life Quality Index (DLQI), and Cardiff Acne Disability Index (CADI) questionnaires were used to assess the effect of acne on quality of life [44,45].

**Figure 5:**
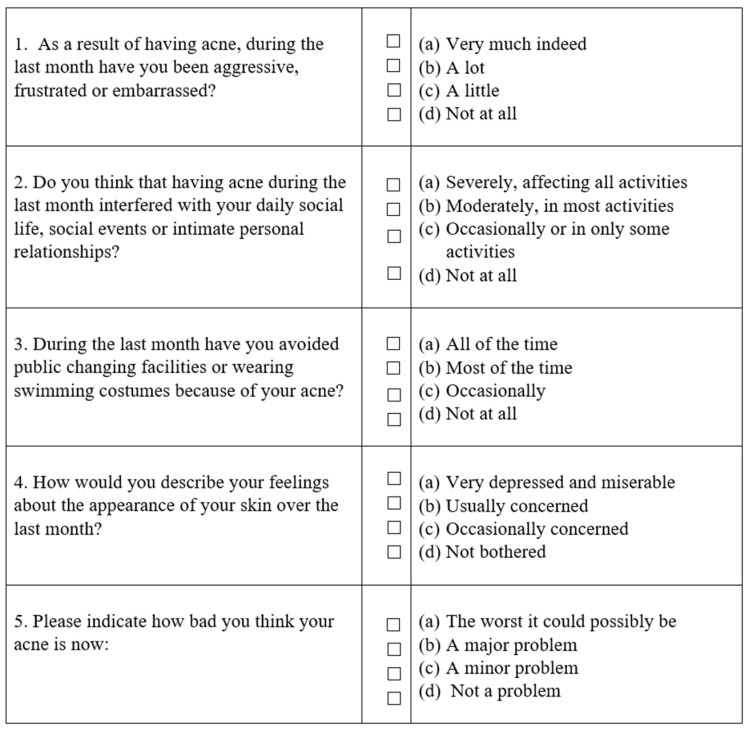
Cardiff Acne Disability Index (CADI).

CADI (Figure 5) is a questionnaire used to evaluate the effect of acne on quality of life. It is characterized by a possible maximum score of 15 and a minimum of 0 (grade of impairment: 0 no impairment, 1–5 mild impairment, 6–10 moderate impairment, and 11–15 severe impairment). The higher the score, the more the quality of life is impaired. 

**Figure 6:**
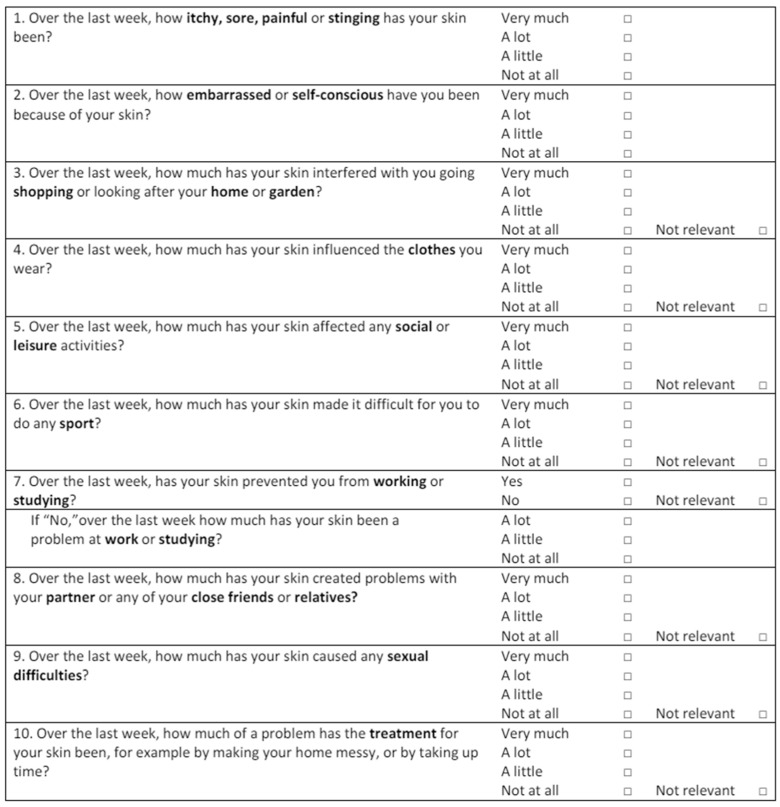
Dermatology Life Quality Index (DLQI).

DLQI (Figure 6), first introduced by Finlay and Khan in 1994 [47], is a ten items tool that evaluates the general effect of skin disease on quality of life. The total score ranges from 0 (zero impact of skin disease on quality of life) to 30 (higher implications for quality of life). The grades are as follows: 0–1, no effect at all on patient’s life; 2–5, a small effect; 6–10, moderate; 11–20, very large; 21–30, extremely large impact on patient’s life. Participants were asked to fill in the DLQI questionnaire without assistance. An English version of the DLQI was translated into Italian by two bilinguals. 

Secondary outcomes were the evaluations of the BMI, hormonal (LH, FSH, testosterone, free testosterone, DHEAS) and metabolic profile (basal insulin, HOMA index), and any associated hirsutism before and after treatment in the same patients.

Hirsutism was quantified with the Ferriman and Gallwey scoring system introduced in 1961 [48]. This system incorporates eleven androgen dependent sites: chest, lip, chin, upper abdomen, lower abdomen, upper arm, forearm, thigh, lower leg, upper back, and lower back. It evaluates eleven different body parts, with scores ranging from zero (no excessive terminal hair growth visible) to four (extensive hair growth visible) for each part of the body evaluated. Thirty-six is the maximum score possible despite that a score of ≥8 typically indicates hirsutism [49]. 

### 4.4. Assessment of Safety and Tolerability

Safety and tolerability were evaluated using continuous monitoring over the treatment period to evaluate the clinical safety of the supplement and to detect any adverse events. The occurrence of adverse effects and compliance were monitored using a diary sheet organized in tables with the opportunity for participants to indicate their assumptions of whether they were undergoing nutraceutical treatment and eventual side effects.

### 4.5. Statistical Analysis

Personal data, in addition to physiological/pathological anamnesis, were collected only at the enrolment visit (T0). The treatment compliance data were collected in T1 and T2. 

Data were systematically entered into an electronic sheet (Excel 2023, Microsoft 2023, Windows 2003, Redmond, WA, USA) throughout the study period. The entries underwent a double check for errors and were subsequently processed using GraphPad Prism 8.0.2 software for Windows. The normality of data distribution was assessed using both the Shapiro-Wilk test and visual inspection via histograms and Q-Q plots. A *p*-value > 0.05 was considered indicative of a normal distribution. All the data showed a normal distribution and were presented as mean ± standard deviation (SD). The demographic and clinical characteristics of the patients were analyzed using one-way analysis of variance (ANOVA), followed by Bonferroni’s test. A significance level of < 0.05 was deemed statistically significant for all conducted tests. A 95.00%Confidence Interval of difference [95.00% CI of diff.] between T1, T2 and T0 was also reported.

## 5. Conclusions

In conclusion, the supplementation of myo-inositol, associated with a microlipodispersed magnesium in a bioaccessible form, and folic acid (LEVIGON™) proved to be extremely useful in reducing insulin resistance, acne, and hirsutism in patients suffering from microcystic ovaries. This treatment was without side effects, thus confirming an excellent compliance. Further double blind, long-term, randomized, clinical trials are needed to confirm this preliminary evidence.

## Figures and Tables

**Table 1:** Hormonal and metabolic profile at baseline, after 3- and 6- months of myo-inositol treatment. Baseline = T0; 3 months = T1; 6 months = T2; One-way ANOVA, followed by Bonferroni’s post test were used for calculating the statistical analysis. Asterisks indicate the *p* value: *** *p* < 0.0001. 95.00% CI of diff. are calculated in comparison with T0.

	T0 (*N* = 200)	T1 (*N* = 200) [95.00% CI of Diff]	T2 (*N* = 200) [95.00% CI of Diff]	*p* Value
Luteinising hormone (LH) (mIU/mL)	15.1 ± 5.1	10.5 ± 3.6 *** [3.7–5.4]	8.3 ± 2.2 *** [5.9–7.6]	<0.0001
Follicle-stimulating hormone (FSH) (mIU/mL)	6.1 ± 1.7	5.5 ± 1.5 *** [0.26–0.93]	4.4 ± 1.3 *** [1.3–2.04]	<0.0001
Testosterone (ng/dL)	103 ± 35	79 ± 29 *** [17.4–30.6]	59 ± 24 *** [37.4–50.6]	<0.0001
Free testosterone (ng/dL)	1.3 ± 0.2	0.8 ± 0.3 *** [0.45–0.55]	0.6 ± 0.2 *** [0.64–0.75]	<0.0001
Dehydroepiandrosterone-sulfate (DHEAS) (mcg/dL)	384 ± 56	251 ± 39 *** [123.4–142.6]	167 ± 31 *** [207.4–226.6]	<0.0001
Basal insulin (mcIU/mL)	25 ± 4,1	22 ± 3.9 *** [3.2–3.8]	17 ± 2.8 *** [7.2–8.8]	<0.0001
HOMA index	2.9 ± 0.7	1.8 ± 0.6 *** [0.9–1.2]	1.4 ± 0.4 *** [1.4–1.6]	<0.0001

**Table 2:** Global Acne Severity, Ferriman-Gallwey score, Cardiff Acne Disability Index and Dermatology Life Quality Index after 3- and 6-months of myo-inositol treatment. Baseline = T0; 3 months = T1; 6 months = T2; One-way ANOVA, followed by Bonferroni’s post test were used for calculating the statistical analysis. Asterisks indicate the *p* value: *** *p* < 0.0001. 95.00% CI of diff. are calculated in comparison with T0.

	*N*	T0	T1	T2	*p* Value
Global Acne Severity (GEA)	200	3.4 ± 1.2	2.1 ± 0.9 *** [1.1–1.5]	0.7 ± 0.6 *** [2.4–2.9]	<0.0001
Ferriman-Gallwey score	60	14.3 ± 3.6	12.1 ± 2.1 *** [1.2–3.2]	8.4 ± 1.2 *** [4.8–6.9]	<0.0001
Cardiff Acne Disability Index (CADI)	200	9.2 ± 1.7	6.1 ± 1.1 *** [2.8–3.4]	2.9 ± 0.9 *** [6.1–6.6]	<0.0001
Dermatology Life Quality Index (DLQI)	200	9.8 ± 1.7	5.2 ± 1.3 *** [4.3–4.9]	2.0 ± 0.8 *** [7.5–8.1]	<0.0001

**Table 3:** BMI after 3- and 6-months of myo-inositol treatment. Baseline = T0; 3 months = T1; 6 months = T2; One-way ANOVA, followed by Bonferroni’s post test were used for calculating the statistical analysis. Asterisks indicate the *p* value: * *p* < 0.05 *** *p* < 0.0001. 95.00% CI of diff. are calculated in comparison with T0.

	*N*	T0	T1	T2	*p* Value
BMI	200	26.5 ± 3.2	25.7 ± 2.9 * [0.14–1.4]	23.9 ± 2.8 *** [1.9–3.3]	<0.0001

**Table 4:** Global Acne Severity (GEA) scale (Adapted from: Schlessinger J et al. J Drugs Dermatol. 2007;6: 607–615.11 [46]).

Score	Severity	Description
0	Clear, No lesions	Residual pigmentation Erythema
1	Almost clear, almost no lesions	A few scattered open or closed comedones Very few papules
2	Mild	Easily recognizable Involve less than half of the face A few open or closed comedones A few papules and pustules
3	Moderate	Involve more than half of the face Many open or closed comedones Many papules and pustules May see one nodule
4	Severe	Involve entire face Many open or closed comedones Many papules and pustules Rare nodules
5	Very severe	Highly inflammatory acne covering the face Presence of nodules

## Data Availability

Data are contained within the article.
